# The Use of Beer Bottles for Samples of Urine

**Published:** 1897-01

**Authors:** 


### THE USE OF BEER BOTTLES FOR SAMPLES OF URINE.

PHILADELPHIA, November 14th, 1896.

*To the Pennsylvania Bottlers' Protective Association of Philadelphia :*

GENTLEMEN :—As you are the people to appeal to against the use of bottles for any other purpose but what they are intended for such as beer, porter, etc., and being in a position to know that they are used over again for other purposes, I take the liberty to request you to emphatically notify the public through our newspapers that the law would be rigidly enforced, should it be violated.

We have occasion, through analytical work, for urine, to get urine from people and sick rooms for such work, and it is a surprising fact that fifty per cent. of the urine brought to us is in beer bottles, which is certainly repugnant to any person of culture or education ; certainly people who do this are not cognizant of the fact that those bottles may come back on their own table.

So, for the sake of all of us, we would request that this universal practice should be fought until it is eradicated.

Hoping you will interest yourself in this matter you will oblige the public at large and some that are interested in the health of the community.

N. B.—Make a canvass of all the physicians in town and see how many bottles of that kind are brought to them by these thoughtless people. * * * *

(Copy of Letter.)

